# The Networks of Noncoding RNAs and Their Direct Molecular Targets in Myocardial Infarction

**DOI:** 10.7150/ijbs.69671

**Published:** 2022-05-01

**Authors:** Shiqi Wang, Yang Wang, Hongxin Cheng, Qing Zhang, Chenying Fu, Chengqi He, Quan Wei

**Affiliations:** 1 Rehabilitation Medicine Center and Institute of Rehabilitation Medicine, West China Hospital, Sichuan University, Chengdu, Sichuan, PR China.; 2 Key Laboratory of Rehabilitation Medicine in Sichuan Province, Chengdu, Sichuan, PR China.; 3 National Clinical Research Center for Geriatrics, West China Hospital, Sichuan University, Chengdu, Sichuan, PR China.; 4 Aging and Geriatric Mechanism Laboratory, West China Hospital, Sichuan University, Chengdu, Sichuan, PR China.

**Keywords:** ncRNAs, miRNAs, lncRNAs, circRNAs, myocardial infarction, data-driven

## Abstract

Noncoding RNAs are closely related to the development of myocardial infarction (MI), and their specific roles in MI are still being carefully studied. Researchers can select the literature they are interested in according to their own wishes in traditional reviews, which results in a certain amount of selection bias. A data-driven approach was used to organize this review to understand the ncRNAs in MI in the past five years. Here, we reveal important networks of interactions between noncoding RNAs and their direct targets. Our review gives an unbiased description of the role of noncoding RNAs in MI. Key information, such as carrier selection, treatment time window, treatment dose and possible side effects of ncRNA therapy, needs to be further determined. In short, the interactions between coding and noncoding genes play indispensable roles in the occurrence and development of MI and still deserve great attention from researchers in this field. The rational application of ncRNAs is expected to become a target for the treatment of MI.

## Introduction

Noncoding RNAs (ncRNAs), which account for approximately 99% of the human genome, can be transcribed but do not encode proteins and have been proven to be related to a variety of human diseases 1. Myocardial infarction (MI) is the most severe type of coronary artery disease and one of the leading causes of death in human beings today 2. In recent years, many studies have been looking for effective strategies for targeted treatment of MI. An increasing number of studies have suggested that ncRNAs, especially microRNAs (miRNAs) and long noncoding RNAs (lncRNAs), are closely related to the development of MI, and their specific roles in MI are still being carefully studied. From this perspective, it is essential and desirable to understand the relationship between ncRNAs and their direct targets, as well as their regulatory effects on the pathophysiology after MI.

The main approach of ncRNA research is to screen one or more ncRNAs from clinical genomics, then search for the corresponding molecular targets and explore their relationships with disease phenotypes. In recent years, many traditional reviews of ncRNAs and MI have been published. However, researchers can select the literature they are interested in according to their own wishes in traditional reviews, which results in a certain amount of selection bias 3, 4. Therefore, a data-driven approach was used to organize this review to understand the ncRNAs in MI in the past five years and to discuss the characteristics of ncRNAs and their molecular targets objectively 5.

Previous researchers explored the networks of ncRNAs and their molecular targets in breast cancer in a similar approach 6. This paper referred to their screening methods and retrieved all the literatures related to ncRNAs (especially miRNAs) and MI in PubMed in the past five years (**Table S1**). Then, the literatures were screened according to the characteristics of the basic research in MI (**Figure S1**). The details of the molecules in the networks are shown in **Table 1**. Finally, the networks of ncRNAs and their direct molecular targets were constructed, and a network is defined as an independent cluster of at least four molecules. Boxes and white ellipses represent ncRNAs and coding genes, respectively, lines with flat red arrows indicate negative interactions or inhibitions, while black arrows indicate positive interactions or activations, and solid lines represent direct effects, while dotted lines represent indirect effects.

In this review, the networks and their components and interactions are discussed, a deeper understanding is gained of the effects of ncRNAs on MI and related mechanisms, and the prospects of the clinical application of ncRNA therapy and its challenges are discussed. It is worth noting that some studies not included in the networks will be referenced for accurate descriptions and discussions to comprehensively understand the overall situation of each ncRNA in the networks.

## The miR-21 PTEN AZIN2-sv/miR-214 network

**Figure 1:**
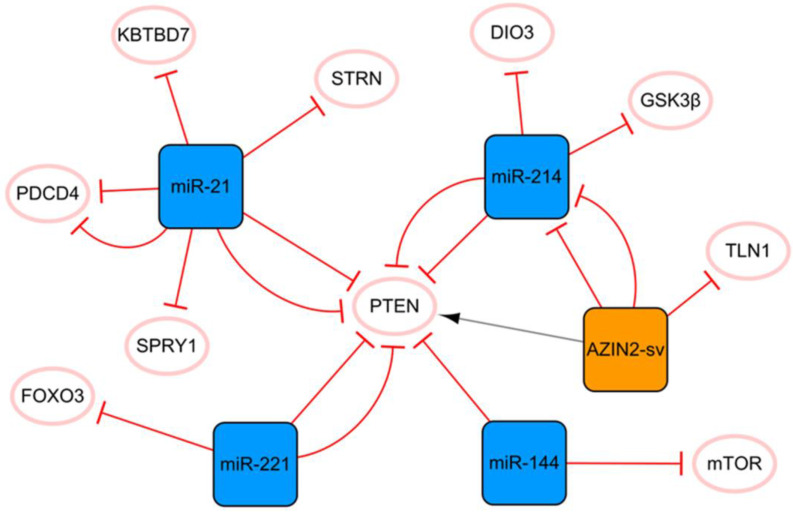
The miR-21 PTEN AZIN2-sv/miR-214 network. The orange box represents lncRNA, the blue boxes represent miRNAs, and the white ellipses represent the protein coding genes. The lines with flat red arrows indicate negative interactions or inhibitions, while black arrows indicate positive interactions or activations, and the solid lines represent direct effects.

PTEN is an essential molecular hub that mediates apoptosis, proliferation, migration, fibrosis, and angiogenesis after MI 7. **Figure 1** shows that PTEN plays a crucial role in this network, connecting miR-21 to the cluster composed of AZIN2-SV and miR-214. MiR-21 plays a pivotal role in cardiomyocyte apoptosis, myocardial fibrosis, and inflammation after MI. MiR-21 enhances the inhibition of cardiomyocyte apoptosis through the PTEN/Akt-P-P38-caspase-3 signaling pathway 8. MiR-21 can also inhibit PDCD4 expression and reduce cell apoptosis 9, 10, and the overexpression of miR-21 can significantly inhibit the release of MI-induced inflammatory cytokines. Bejerano T et al. found that when nanoparticles of miR-21 mimics were delivered to cardiac macrophages, the level of the anti-inflammatory gene SOCS1 was significantly increased, suggesting that the increase in the expression level of miR-21 in cardiac macrophages after MI could lead to a change in phenotype from pro-inflammatory to anti-inflammatory 11. Moreover, miR-21 targets KBTBD7 and inhibits the activation of the P38 and NF-κB signaling pathways directly, thereby reducing inflammation after MI 12. However, the upregulation of miR-21 does not bring full benefits because it targets SPRY1 to inhibit the activation of the ERK signal, thus promoting myocardial fibrosis 13. The authors found that miR-21 knockout mice developed more pronounced cardiac fibrosis, as did *in vitro* studies based on primary cardiac fibroblasts. Under hypoxic conditions, the expression of miR-21 is significantly upregulated, and it targets and downregulates the level of STRN and regulates the expression of NOS3, resulting in the disorder of nitrogen metabolism 14. Currently, a specific anti-miR-21 has been demonstrated to effectively improve cardiac remodeling in a porcine MI model 15. However, inhibition of miR-21 inactivates the profibrotic pathway, which is not conducive to the repair of infarction scars and may even prevent the body from inhibiting myocardial cell apoptosis 16. Therefore, although the regulation of miR-21 expression is a potential method, its application in clinical practice still has limitations.

AZIN2-sv and miR-214 form a cluster with a clear targeting relationship. AZIN2-sv can inhibit angiogenesis by promoting ubiquitin-dependent degradation of TLN1 after MI 17. AZIN2-sv acts as a miR-214 sponge to release PTEN, blocks the activation of the PI3K/Akt signaling pathway, and inhibits cardiomyocyte proliferation 18. In addition, we know that lncRNAs in the cytoplasm regulate signaling by interacting with specific proteins. Li X et al. identified relevant proteins interacting with AZIN2-sv in cardiomyocytes by an RNA pull-down assay and found that AZIN2-sv could increase the stability of PTEN through direct binding 18. Previous studies have shown that GSK3β inhibition has strong angiogenic and anti-apoptosis effects and plays an important role in endothelial angiogenesis 19. The upregulation of miR-214 can inhibit the expression of Gsk3β and promote the activity of β-catenin, thus promoting cardiac regeneration and functional recovery after MI 20. It is worth mentioning that triiodothyronine (T3) is an essential regulator of cardiac contractility and metabolism. DIO3 can convert T3 into diiodothyronine (3,3′-T2). The upregulation of miR-214 leads to a decrease in DIO3, which limits the reduction in cardiac T3 signaling and protects cardiac systolic function 21. It is not difficult to see that miR-214 plays a protective role in MI, and most of the current evidence shows that miR-214 has a positive effect on the cellular behavior of cardiomyocytes and endothelial cells. However, it has also been found that overexpression of miR-214 in cardiomyocytes may induce dilated cardiomyopathy. This again suggests that the dosage and timing of ncRNA therapy are critical.

In this network, two miRNA axes are also strung through PTEN, namely, miR-221 and miR-144. Circulating miR-221 has been shown to be increased in patients with AMI, and many studies have confirmed that upregulated miR-221 from different sources can improve angiogenesis and cell proliferation and inhibit apoptosis through the PTEN/Akt pathway 22, 23. In addition, the upregulation of miR-221 negatively regulates FOXO3, thereby inhibiting ATG7 transcription and reducing the occurrence of fibrosis after MI 24. The level of serum miR-144 was significantly correlated with MI and was found to be highly expressed in the infarcted area of the porcine MI model. Compared with the function of miR-221 in alleviating myocardial fibrosis, there is some contradiction in the phenotype of miR-144 in myocardial fibrosis. It was reported that miR-144 enhanced myocardial fibrosis after MI by targeting PTEN 25. However, Li J et al. reported that the increase in miR-144 decreased the size of infarction and collagen scarring, and miR-144 could regulate fibrosis signaling at least in part by downregulating TGF-β signaling 26. For such different results, we think that the main reason may be related to the different animal models used in the two studies (Yuan X et al. established the MI model in pigs, while Li J et al. established in mice). There are great differences in structure, heart rate and oxygen consumption between mouse hearts and human hearts, while pig hearts are closer to human heart, especially in terms of their coronary distribution characteristics, and both have less collateral circulation 27. Therefore, more rigorous preclinical studies are needed to treat miR-144 as a therapeutic target for MI. In addition, cardiomyocytes are long-lived postdivision cells with little ability to differentiate and regenerate. Therefore, autophagy plays a vital role in maintaining cardiac function and vitality. MTOR is a crucial negative regulator of the autophagy pathway, and miR-144 can inhibit mTOR from enhancing autophagy signaling, which may also be related to cardiac protection 26.

## The MALATA1/miR-92a network

**Figure 2:**
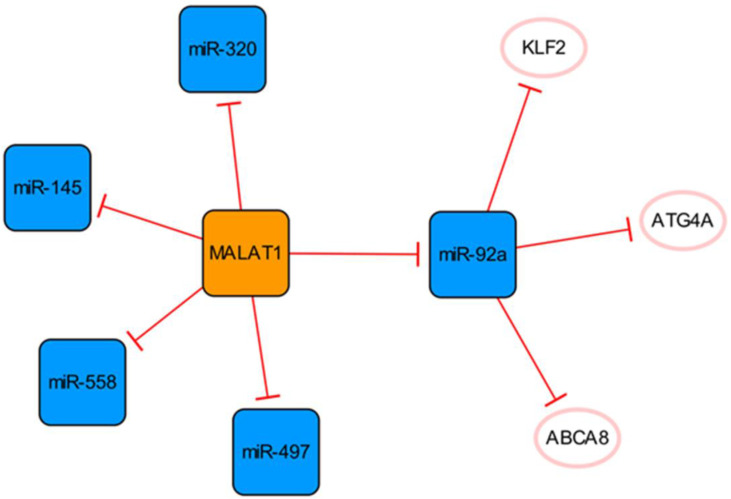
The MALATA1/miR-92a network. The orange box represents lncRNA, the blue boxes represent miRNAs, and the white ellipses represent the protein coding genes. The lines with flat red arrows indicate negative interactions or inhibitions, and the solid lines represent direct effects.

This subnetwork is primarily constructed by connecting MALAT1 and miR-92a, and there is a clear targeting relationship between them (**Figure 2**). Upregulation of MALAT1 can inhibit the expression of miR-92a in the left ventricular myocardium after MI and indirectly promote the expression of KLF2, thus promoting neovascularization 28.

The expression of MALAT1 is increased in patients with MI and has been confirmed to activate the hypoxia pathway 29, 30. MALAT1 can be used as a sponge to regulate the expression of miR-145 and promote myocardial fibrosis mediated by the TGF-β1 pathway 29. MALAT1 can inhibit apoptosis by targeting miR-497 31. However, MALAT1, as the competing endogenous RNA (ceRNA) of miR-320, regulates the expression of PTEN in mouse cardiomyocytes. Through the miR-320/PTEN axis, overexpression of MALAT1 can promote cardiomyocyte apoptosis 32. Although Wu Q et al. identified the binding of MALAT1 to miR-497, they did not know how much MALAT1 needed to be delivered to the infarcted heart to fully bind and inhibit miR-497. Meanwhile, their findings were at the cellular level, and their studies used neonatal rat cardiomyocytes, which did not fully reflect the final results of adult cardiomyocytes 31. They also admitted that the cardioprotective effect and potential mechanism of MALAT1 need to be verified in infarcted hearts, while Hu H et al. explored a lncRNA MALAT1 gene knockdown in post-MI mice 31, 32. Furthermore, miR-558 is also a direct target of MALAT1, which can protect cardiomyocytes from isoproterenol-induced apoptosis by enhancing UlK1-mediated protective autophagy through sponging miR-558 33. The function of the MALAT1 was further demonstrated in knockout mice, while the mice disease model was not established by ligation of coronary arteries in the study, but by injection of isoproterenol. Therefore, based on the specific methods of various studies and research results, we consider that it is difficult to determine the specific effect of MALTA1 on cardiomyocytes apoptosis. In addition, the upregulation of miR-497 eliminate the role of MALAT1 in promoting endothelial cell (EC) tube formation, suggesting that the high expression of MALAT1 can promote angiogenesis by inhibiting the expression of miR-497 31.

It is known that an appropriate autophagy level can maintain cell homeostasis and promote cell survival. ECs enhance their ability to use their organelles as fuel substrates by increasing autophagic flux 34. Meanwhile, the arrangement of ECs under physiological blood flow also depends on autophagy 35. MiR-92a targets and inhibits the expression of ATG4A and limits endothelial autophagy. In addition, miR-92a targets ABCA8B and CD36, interfering with cholesterol transport, reducing the level of high-density lipoprotein, and limiting myocardial fatty acid uptake 34. In conclusion, high expression of miR-92a will aggravate injury after MI, and targeted inhibition of miR-92a expression can be used as a new therapeutic option, which has clinical application value in alleviating tissue damage in patients with MI.

## The MEG3/miR-125b/miR-26a network

**Figure 3:**
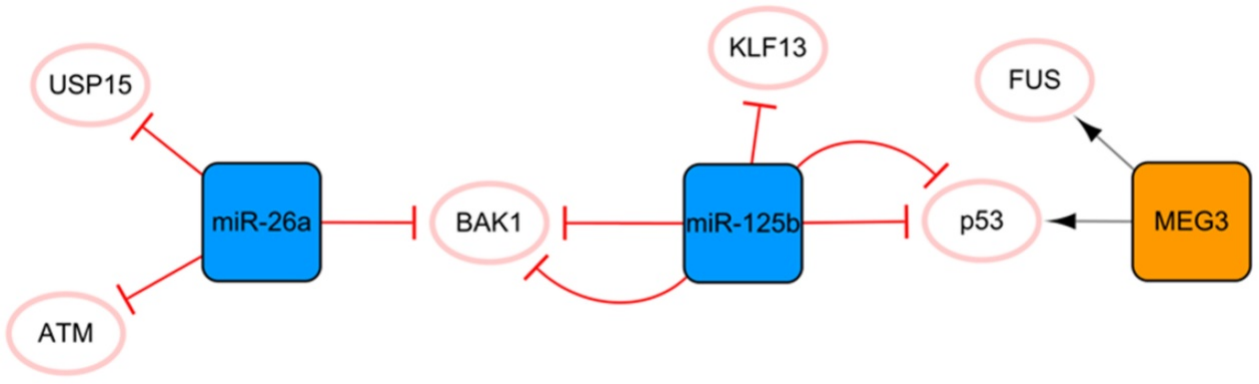
The MEG3/miR-125b/miR-26a network. The orange box represents lncRNA, the blue boxes represent miRNAs, and the white ellipses represent the protein coding genes. The lines with flat red arrows indicate negative interactions or inhibitions, while black arrows indicate positive interactions or activations, and the solid lines represent direct effects.

This network is closely associated with apoptosis (**Figure 3**). MEG3 transcribed by imprinted genes is a lncRNA that is significantly related to apoptosis 36. Under hypoxic conditions, MEG3 can be directly upregulated by p53 and participate in the regulation of apoptosis through direct binding to the RNA binding protein FUS 36. Moreover, MEG3 knockout can inhibit the expression of p53 and reduce the apoptosis induced by endoplasmic reticulum stress after MI 37. Connected by p53 is another protagonist in this subnetwork, miR-125b, which is also significantly related to apoptosis. MiR-125b has antiapoptotic effects on ischemic cardiomyocytes *in vivo* and *in vitro* by inhibiting p53 and BAK1 38. MiR-125b acts as a protective miRNA by inhibiting pro-apoptotic KLF13 in cardiomyocytes 39. It is well known that the appropriate level of autophagy has a robust protective effect. Blocking autophagy can enlarge the area of MI, but excessive autophagy can promote apoptosis and necrosis 40. Regarding the specific anti-apoptosis mechanism of miR-125b, further studies have found that it interferes with p53/Bnip3 signal transduction, inhibits autophagic flux, and reduces cell death 41. In this subnetwork, miR-26a is connected to miR-125b through the proapoptotic gene BAK1, and miR-26 plays an antiapoptotic role by targeting BAK1 42. Overexpression of miR-26a activates autophagy by targeting USP15, which reduces cardiomyocyte death induced by ischemic stress 43. In addition, ATM is a target for the treatment of MI, and miR-26a can minimize the development of MI by inhibiting the expression of ATM in MI 44. The ncRNA in this network has a consistent conclusion on the regulation of apoptosis after MI, with no obvious contradictory conclusion; therefore, this network may be the preferred network for the target selection of anti-apoptosis strategies of ncRNA therapy.

## The TUG1/miR-124 network

**Figure 4:**
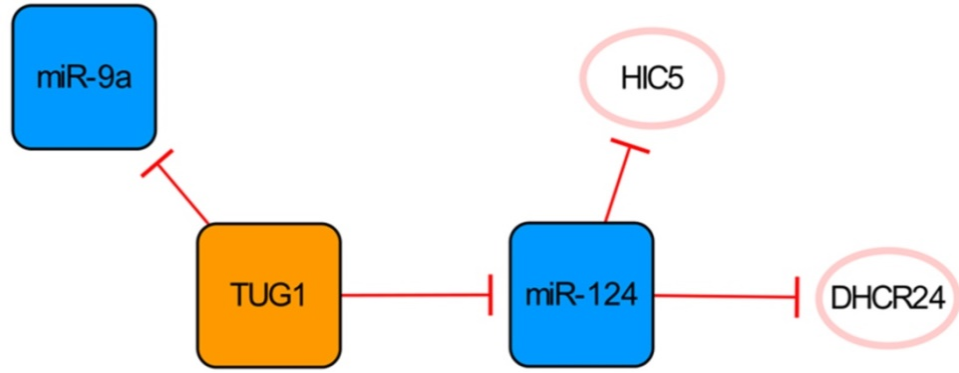
The TUG1/miR-124 network. The orange box represents lncRNA, the blue boxes represent miRNAs, and the white ellipses represent the protein coding genes. The lines with flat red arrows indicate negative interactions or inhibitions, and the solid lines represent direct effects.

LncRNA TUG1 and its two targets constitute a network significantly related to apoptosis (**Figure 4**). Downregulation of the lncRNA TUG1 gene significantly improves cardiac function in mice with MI. MiR-9a exerts an anti-cardiomyocyte apoptosis effect by inhibiting KLF5. As a competitive endogenous RNA of miR-9a, overexpressed TUG1 further aggravates myocardial cell apoptosis by downregulating the expression of miR-9a 45, while conversely, Jiang N et al. have found that TUG1 seems to have anti-apoptotic ability because the overexpression of HIC5 can enhance the viability, migration and invasion of cells induced by hypoxia injury and then reduce the apoptosis of cells 46. MiR-124, one of the direct targets of TUG1, can inhibit the expression of HIC5 and aggravate the damage to cardiomyocytes caused by hypoxia. Therefore, overexpression of TUG1 can downregulate miR-124 and upregulate the expression of HIC5 indirectly, thereby increasing the expression of SP1 and SURVIVIN and reducing cardiomyocyte damage and apoptosis 46. As also shown in this network, DHCR24 is another target of miR-124, and the overexpression of miR-124 can significantly increase cardiomyocyte apoptosis by targeting the inhibition of DHCR24 47. The regulatory functions of miR-9a and miR-124 in this network on apoptosis are completely opposite. Interestingly, they are all direct targets of TUG1, which leads to conflicting results. However, it is worth mentioning that Yang D et al. observed the pro-apoptotic effect of TUG1 on the animal level in their study, which might be more convincing 45.

## The KCNQ1OT1/miR-152 network

**Figure 5:**
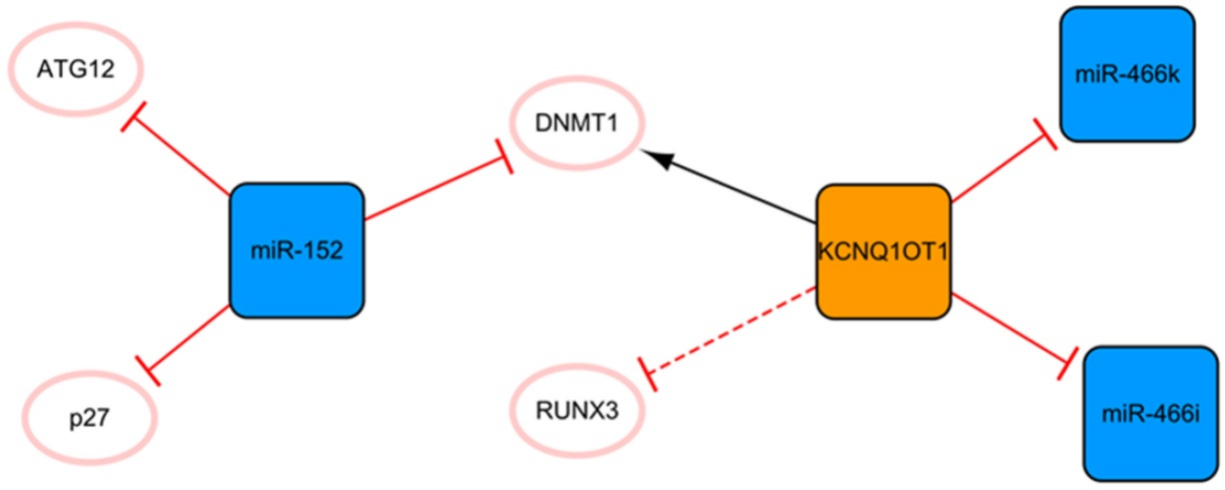
The KCNQ1OT1/miR-152 network. The orange box represents lncRNA, the blue boxes represent miRNAs, and the white ellipses represent the protein coding genes. The lines with flat red arrows indicate negative interactions or inhibitions, while black arrows indicate positive interactions or activations. The solid lines represent direct effects, while dotted lines represent indirect effects.

The network shown in **Figure 5** demonstrates the long noncoding KCNQ1OT1 and miR-152. These ncRNAs are connected through DNMT1. KCNQ1OT1 can promote RUNX3 methylation by recruiting DNMT1, which leads to the downregulation of RUNX3 expression and regulation of cardiac microvascular endothelial cell activity and the inflammatory response in mice after MI 48. After KCNQ1OT1 gene knockout, the proliferation of cardiac microvascular ECs after AMI is promoted, while apoptosis is inhibited, accompanied by a decrease in the level of inflammatory cytokines. These trends can also be realized by RUNX3 overexpression via the Notch pathway 48. Overexpression of KCNQ1OT1 can also target miR-466k and miR-466i to play sponge roles and trigger cardiomyocyte injury in the process of MI 49. At present, the role of KCNQ1OT1 after MI is not known, so the inhibition of KCNQ1OT1 as the target may be a potential direction of ncRNA therapy. There are relatively fewer studies on miR-152 in MI, and only two articles studying it were retrieved using our search methods; however, miR-152 is expected to become a new target for MI therapy in the future. MiR-152 inhibits the expression of DNMT1 and the cell cycle inhibitory protein p27, leading to cardiomyocyte proliferation 50. In addition, a luciferase reporter assay confirmed that miR-152 targets ATG12, suggesting that it might have a potential antiapoptotic effect mediated by regulating autophagy 51. Of course, this also needs to be proven by subsequent experiments.

## The miR-590/miR-199a network

**Figure 6:**
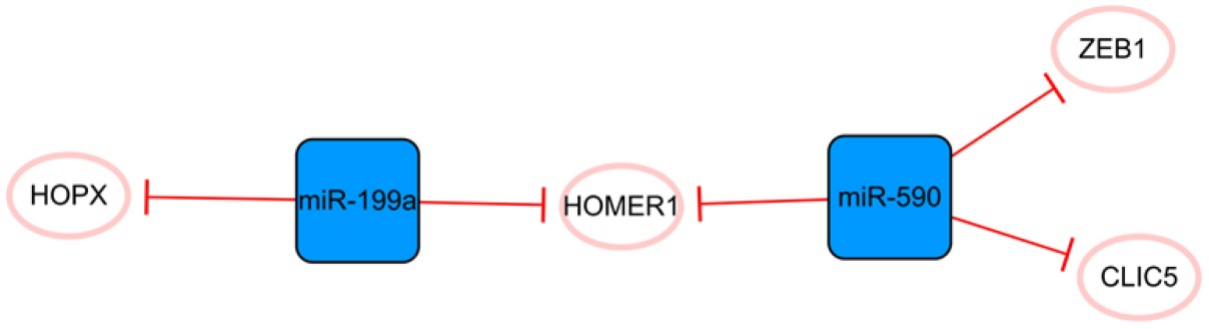
The miR-590/miR-199a network. The blue boxes represent miRNAs, and the white ellipses represent the protein coding genes. The lines with flat red arrows indicate negative interactions or inhibitions, and the solid lines represent direct effects.

This network consists only of miRNAs strung by HOMER1 (**Figure 6**). Some studies have demonstrated that miR-199a and miR-590 can promote the proliferation of cardiomyocytes, which may be due to their cumulative effects on multiple cellular mRNA targets 52. The common target HOMER1 is involved in the regulation of calcium signaling in cardiomyocytes and HOPX, and the direct target of miR-199a is a key negative regulator of cardiomyocyte proliferation 53-55. CLIC5 is also a direct target of miR-590, but its role in MI is not completely clear 52. It is only known that CLIC5 plays a direct role in regulating the production of mitochondrial reactive oxygen species and is associated with the actin-based cytoskeleton 56, 57. In brief, miR-199a and miR-590 inhibit the expression of these functional molecules, produce cumulative effects and stimulate the proliferation of cardiomyocytes 52, 55. Lesizza P et al. further found that viral vector-based expression of miR-199a and miR-590 in mouse hearts could induce cardiac regeneration after MI 58. In addition, miR-590 has been found to significantly inhibit the proliferation and migration of human cardiac fibroblasts and reduce the mRNA and protein expression levels of α-SMA, Col1A1, and Col3A 59. The specific mechanism is that miR-590 directly targets the 3'UTR of ZEB1, thus inhibiting the translation of ZEB1 59. Interference with the expression of ZEB1 decreases cell proliferation, migration activity, and the expression of the abovementioned related proteins. Overall, this network is closely associated with cell proliferation, and the targeting relationship between each molecule is relatively clear. The overexpression of miRNAs in this network can promote the proliferation of cardiomyocytes and effectively inhibit the proliferation and transformation of fibroblasts.

## The miR-155 network

**Figure 7:**
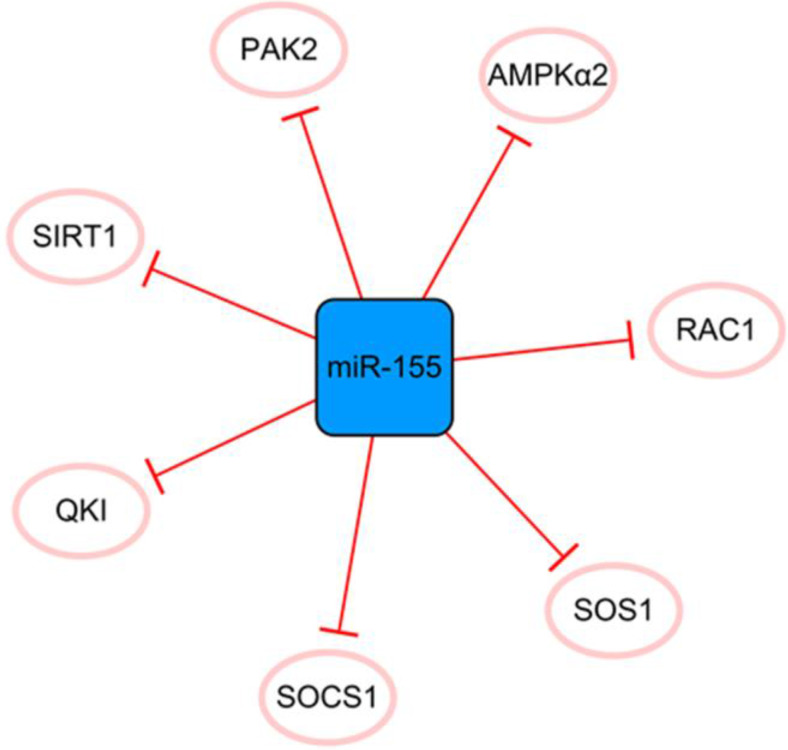
The miR-155 network. The blue box represents miRNA, and the white ellipses represent the protein coding genes. The lines with flat red arrows indicate negative interactions or inhibitions, and the solid lines represent direct effects.

MiR-155 has many direct targets that constitute a separate network (**Figure 7**). MiR-155 inhibits the proliferation of fibroblasts and alleviates fibrosis after MI to some extent 60. Fibroblast proliferation may not bring complete harm to the infarcted heart. The extracellular matrix secreted by fibroblasts after activation can effectively promote the healing of the heart after infarction and prevent the occurrence of ventricular rupture; therefore, it is crucial to effectively control the expression of miR-155 61. However, the disadvantages of miR-155 overexpression far outweigh its contribution to ameliorating fibrosis after MI. It was reported that miR-155 could promote MI-induced apoptosis by targeting QKI 60. Furthermore, the level of SOCS1 in cardiac fibroblasts is decreased by miR-155 mimics and promotes inflammation 62. It inhibits angiogenesis and exacerbates cardiac dysfunction by downregulating its target genes, including RAC1, PAK2, SIRT1, and AMPKα2 63. It can be seen that if we want to achieve the goal of treating MI with miR-155 as a regulatory target, it is necessary to regulate the specific expression of miR-155 in different target cells and define its specific treatment time window. In general, miR-155 is like a double-edged sword, and reasonable application is expected to become a target for the treatment of MI.

## The circHIPK3 network

**Figure 8:**
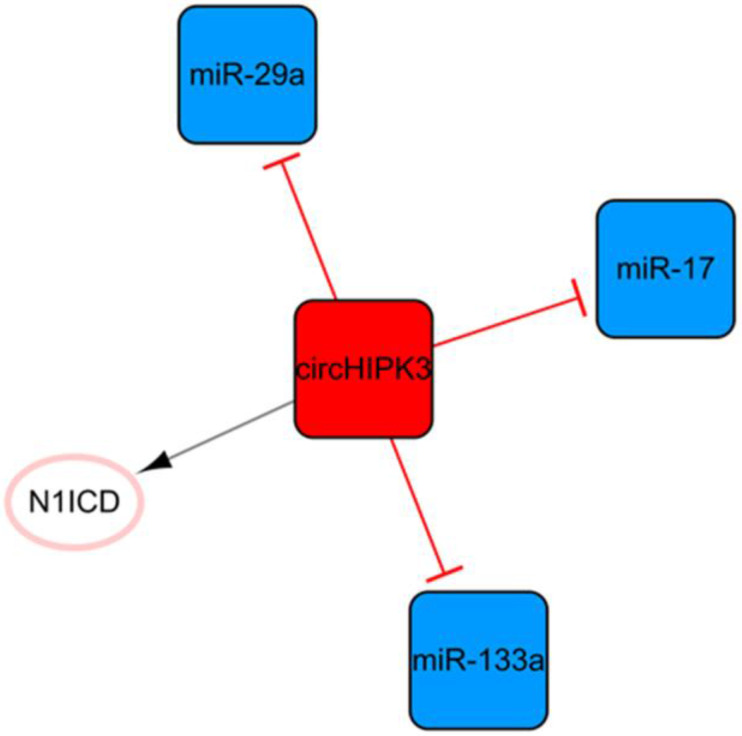
The circHIPK3 network. The red box represents circRNA, the blue boxes represent miRNAs, and the white ellipses represent the protein coding genes. The lines with flat red arrows indicate negative interactions or inhibitions, while black arrows indicate positive interactions or activations, and the solid lines represent direct effects.

Circular RNA (circRNA) is another kind of ncRNA that is mainly produced by the back-splicing of exons and covalent bonds 64. CircHIPK3, the only circRNA in this ncRNA network, is transcribed from the second exon of HIPK3 and plays pivotal roles in cell growth and angiogenesis **(Figure 8)** 65, 66. CircHIPK3-rich exosomes can be released during hypoxia in cardiomyocytes. CircHIPK3 can increase the expression of VEGFA by inhibiting the expression of miR-29a, thus promoting the cell cycle progression and proliferation of cardiac ECs and changing the proliferation, migration, and tube formation of cardiac ECs 67. Meanwhile, circHIPK3 can act as a sponge of miR-133a as well, indirectly promoting the expression of connective tissue growth factors, activating ECs, and improving angiogenesis after MI 68. CircHIPK3 enhances the stability of N1ICD by acetylation and promotes the proliferation of cardiomyocytes 68. In addition, the upregulation of circHIPK3 expression after epinephrine administration can temporarily improve cardiac function by increasing the peak concentration of Ca^2+^ in cardiomyocytes via the circHIPK3/miR-17/ADCY6 axis 69. However, downregulation of circHIPK3 can alleviate fibrosis after MI in mice and maintain cardiac function 69. This means that short-term upregulation of circHIPK3 can temporarily improve cardiac function; while long-term high expression may aggravate cardiac fibrosis and decrease cardiac function.

## Other lncRNA/miRNA networks

**Figure 9:**
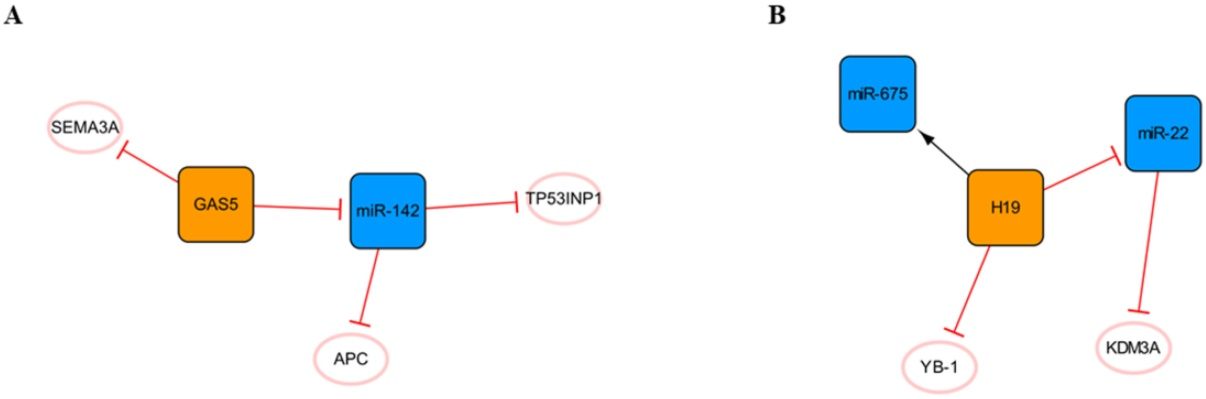
Other lncRNA/miRNA networks. **(A)** The GAS5/miR-142 network. **(B)** The H19/miR-22 network. The orange boxes represent lncRNAs, the blue boxes represent miRNAs, and the white ellipses represent the protein coding genes. The lines with flat red arrows indicate negative interactions or inhibitions, while black arrows indicate positive interactions or activations, and the solid lines represent direct effects.

In addition to the networks mentioned above, there are also some networks composed of lncRNAs and miRNAs that deserve our attention **(Figure 9)**. The regulatory role of lncRNA GAS5 in cancer cells has been widely reported 70, and it forms a network with miR-142** (Figure 9A)**. RNA immunoprecipitation revealed that GAS5 is the molecular sponge of miR-142. TP53INP1 is the target gene of miR-142. GAS5 silencing promotes the activation of the PI3K/AKT and MEK/ERK signaling pathways in a miR-142-dependent manner and weakens the cardiomyocyte apoptosis induced by hypoxia 71. In short, the high expression of GAS5 may aggravate cardiomyocyte apoptosis, and many other studies have confirmed this point of view 72, 73. However, another study showed that GAS5 could negatively regulate the expression of SEMA3A protein and reduce cardiomyocyte apoptosis 74, and we consider that different sources of cardiomyocytes and modeling methods may lead to two opposing outcomes. In addition, it can be found in this network that APC, a negative regulator of the WNT signaling pathway, is another target of miR-142. It was found that miR-142 could directly target and inhibit the expression of APC and promote the activation of the WNT signaling pathway and cardiac fibroblasts 75.

The H19 network is shown in **Figure 9B**. H19, known as a fetal gene, is downregulated after birth^76^. H19 polymorphism has been shown to be significantly associated with the risk of coronary artery disease, suggesting that H19 plays an important role in cardiac pathogenesis 77. Choong OK et al. found that H19 was gradually upregulated within a week after MI, suggesting that H19 might play a role in the early stage of cardiac remodeling. Subsequent studies on the function of H19 in the early postinfarction period illustrated that the early ectopic expression of H19 in the mouse heart led to severe cardiac dilation and fibrosis. This may be related to the direct interaction of H19 with YB-1 under hypoxia, and the inhibition of YB-1 can promote the expression of COL1A1 and lead to myocardial fibrosis 78. Another study found that miR-22 is the direct target of H19 and that miR-22 targets the inhibition of KDM3A directly. H19 regulates the expression of KDM3A in a miR-22-dependent manner to reduce myocardial injury 79. In addition, H19 in exosomes derived from bone marrow mesenchymal stem cells can be upregulated by the clinically common lipid-lowering drug atorvastatin calcium. The upregulation of H19 can regulate the expression of miR-675, activate VEGF and the mediators of intercellular adhesion molecule-1, promote angiogenesis, and reduce myocardial cell apoptosis 80. Briefly, the high expression of H19 in the early stage after MI can alleviate a series of injuries caused by acute ischemia to a certain extent, promote angiogenesis, reduce myocardial apoptosis, and achieve protective effects. However, every coin has two sides, and high expression of H19 can also promote the occurrence of myocardial fibrosis. Therefore, inhibiting the interaction between H19 and YB-1 while upregulating the high expression of H19 in the early stage of MI may be a therapeutic strategy that is worthy of further study.

## Other miRNA networks

**Figure 10:**
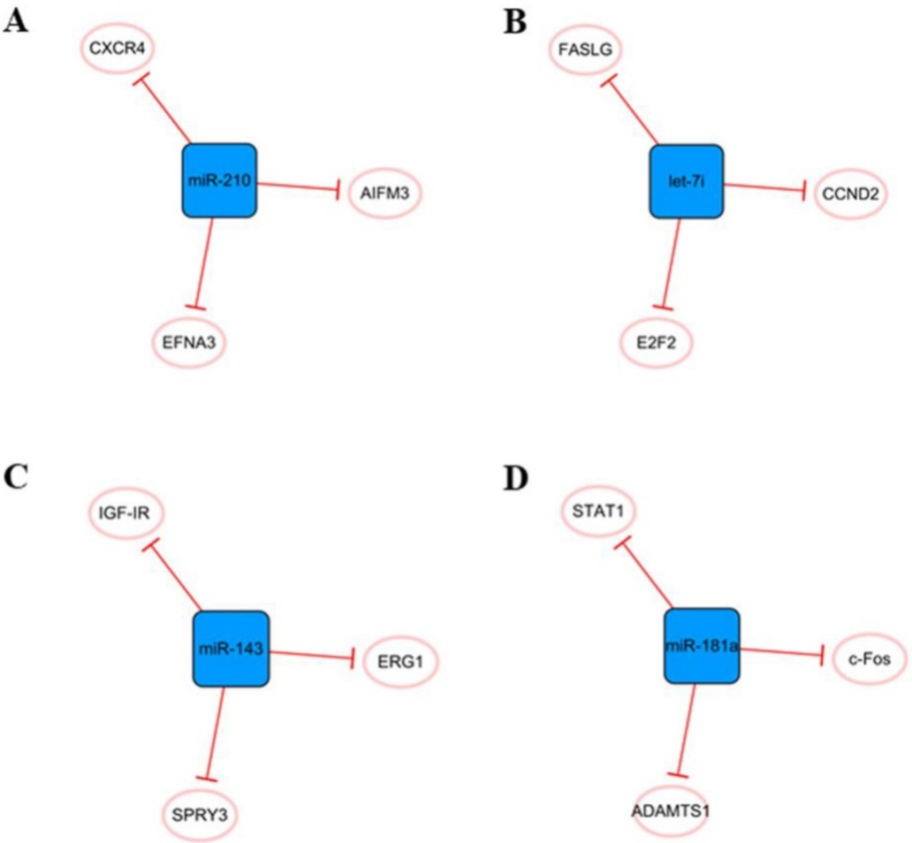
Other miRNA networks. **(A)** The miR-210 network.** (B)** The leti-7i network. **(C)** The miR-143 network.** (D)** The miR-181a network. The blue boxes represent miRNAs, and the white ellipses represent the protein coding genes. The lines with flat red arrows indicate negative interactions or inhibitions, and the solid lines represent direct effects.

Other networks, which consist only of miRNAs and their coding genes, have also attracted the attention of researchers **(Figure 10)**. The main cause of MI is ischemic hypoxia, and miR-210 is considered a ncRNA closely related to hypoxia 81. There is increasing evidence that miR-210 can be used as an independent indicator of MI severity 82. It has been found that miR-210 is upregulated in hypoxic cardiomyocytes, which can alleviate hypoxia-induced damage by targeting CXCR4 inhibition 83. Hypoxia can also induce the upregulation of miR-210 expression in exosomes derived from mesenchymal stem cells. Overexpression of miR-210 inhibits AIFM3, activates PI3K/AKT and p53 signal transduction, reduces apoptosis, and plays a role in cell protection 84. In addition, miR-210 has been found to promote angiogenesis in mice with MI, which may be related to the targeted inhibition of EFNA3 as a protein regulating angiogenesis 85. In short, miRNA-210 has great potential in the treatment of cardiovascular diseases, and it is necessary to further study the cardiovascular protective mechanism of miR-210 and its future applications.

Elevated miR-143 level was detected in human MI samples 86. When the expression of miR-143 is upregulated, it can directly target SPRY3, activate the P38, ERK, and JNK pathways, and promote the proliferation, migration, and transformation of human cardiac fibroblasts and the excessive accumulation of extracellular matrix, thus aggravating the occurrence of myocardial fibrosis 86. Overexpression of miRNA-143 can inhibit IGF-IR, which has a negative effect on angiogenesis 87. Moreover, downregulation of miR-143 can regulate EGR1, improve the dysfunction of I_Na_ and I_K1_ currents in ventricular myocytes, and effectively inhibit ventricular arrhythmias 88. Therefore, downregulation of miR-143 expression after MI seems to be a feasible therapeutic strategy.

The let-7 family is one of the earliest discovered miRNAs and plays an important role in the regulation of cardiovascular disease 89. Let-7i, a member of the let-7 family, is abundant in cardiomyocyte-derived exosomes under hypoxia and has a potential anti-apoptosis effect by targeting FASLG expression 51. In addition, let-7i is involved in the regulation of the cardiomyocyte cell cycle and further affects proliferation, and E2F2 and CCND2 are two direct targets of let-7i. After the inhibition of let-7i, the expression of E2F2 and CCND2 is significantly upregulated, which can promote the proliferation of cardiomyocytes^90^. In fact, the let-7 family is involved in many cellular processes, including proliferation, inflammation and apoptosis 91, but their roles in MI have not been well studied. Therefore, it is necessary to further study their roles in cardiovascular diseases.

We believe that miR-181a may be an effective therapeutic target to reduce inflammation and improve cardiac remodeling after MI. The aldosterone-mineralocorticoid receptor pathway is activated during MI 92. Garg An et al. found that overexpression of miR-181a could significantly inhibit aldosterone-mineralocorticoid-induced cardiomyocyte hypertrophy. The mechanism might be related to the direct targeting of miR-181a to inhibit the expression of ADAMTS1 and then regulate the level of Ngala 93. Moreover, MI was accompanied by severe inflammation, and the expression of miR-181a was found to be increased in dendritic cells. Overexpression of miR-181a can reduce the maturation of dendritic cells and the production of inflammatory cytokines, prevent the occurrence of severe inflammation, and inhibit the apoptosis of cardiomyocytes under hypoxia by inhibiting STAT1 and c-Fos 94.

## Perspectives and challenges

NcRNAs have been well studied in cellular processes and cell types associated with the cardiovascular system 95. After MI, the internal environment of cell survival changes, and the expression of some ncRNAs changes. The expression of different ncRNAs affects the prognosis of MI 96. They provide not only potential biomarkers for the diagnosis of MI but also new possibilities and opportunities for the selection of therapeutic targets 96. Applying ncRNA therapy in the clinic is a very attractive prospect. However, before translating the therapy into the clinic, a large number of preclinical studies are needed to ensure its efficacy and safety in patients with MI. In fact, some ncRNAs in these networks have been subjected to some preclinical studies. MiR-199a can promote the proliferation of endogenous cardiomyocytes to realize heart repair in pigs 97, and the use of miR-144 inhibitors can also reduce the occurrence of fibrosis after MI in pigs 25.

However, there are still some challenges in the clinical application of ncRNA therapy. First, ncRNAs have opposite effects on cells in different tissues. NcRNAs exist widely in different cells, and some are thought to be therapeutic targets for MI, which may aggravate the occurrence and development of other diseases. H19, mentioned above, is a typical example, which reduces cardiomyocyte apoptosis and promotes angiogenesis in the early stage after MI, but overexpression of H19 promotes the invasion, migration and epithelial-mesenchymal transformation of ovarian cancer cells. Second, ncRNAs can have different effects on different cells in the same tissue 98. The cellular composition of heart tissue is complex. In addition to cardiomyocytes, there are many cellular components, such as macrophages, cardiac fibroblasts, and endothelial cells. NcRNAs play different roles in different cells, which make targeted treatment of MI difficult 99. The above mentioned miR-21 plays a key role in anti-cardiomyocyte apoptosis and inflammation after infarction, but its overexpression may promote the proliferation and differentiation of cardiac fibroblasts and aggravate fibrosis after MI. All these conditions increase the clinical complexity and difficulty of the application of ncRNA therapy.

In addition, high-throughput sequencing found that there are a large number of changes in ncRNA levels in cells or tissues under pathological conditions, and repairing the expression levels of single or several kinds of ncRNAs may not necessarily reach the ideal level of treatment. More importantly, some of the effects of ncRNAs have opposite conclusions in the heart, such as the regulation of cardiomyocyte apoptosis by TUG1 and MALAT1 31, 32, 45, 46. This may be related to the selection of cells and targeted regulatory molecules *in vitro*, but large animal experiments are also urgently needed to further determine their specific roles *in vivo*.

Controlling the expression of ncRNAs is also very important for the clinical application of ncRNA therapy. At present, the ncRNA delivery carriers used in some studies are liposomes, which may lead to the overexpression of ncRNAs in the cardiovascular system and produce a nonspecific interferon response 4. Therefore, a new vector, such as adenovirus, can be chosen to avoid ncRNA overexpression *in vivo*. To regulate the specific expression profiles of ncRNAs in different tissues, focusing on the use of different biomedical technologies like nanoparticle-hydrogel systems is also a future research direction 100. Meanwhile, the treatment dose, treatment time window and possible side effects of ncRNA therapy need to be further determined.

## Conclusions

Our review reveals important networks of interactions between ncRNAs and their direct targets and provides an unbiased description of the roles of ncRNAs in MI over the last five years. First, our review manually searched and screened all the literature on noncoding RNAs related to MI in the last five years available on PubMed. Second, only the original studies of ncRNA-targeting relationships or interactions confirmed by laboratory methods *in vitro* and/or *in vivo* could be included. Finally, the relationships between the noncoding RNAs and their direct molecular targets in the networks were discovered and described.

**Figure 11:**
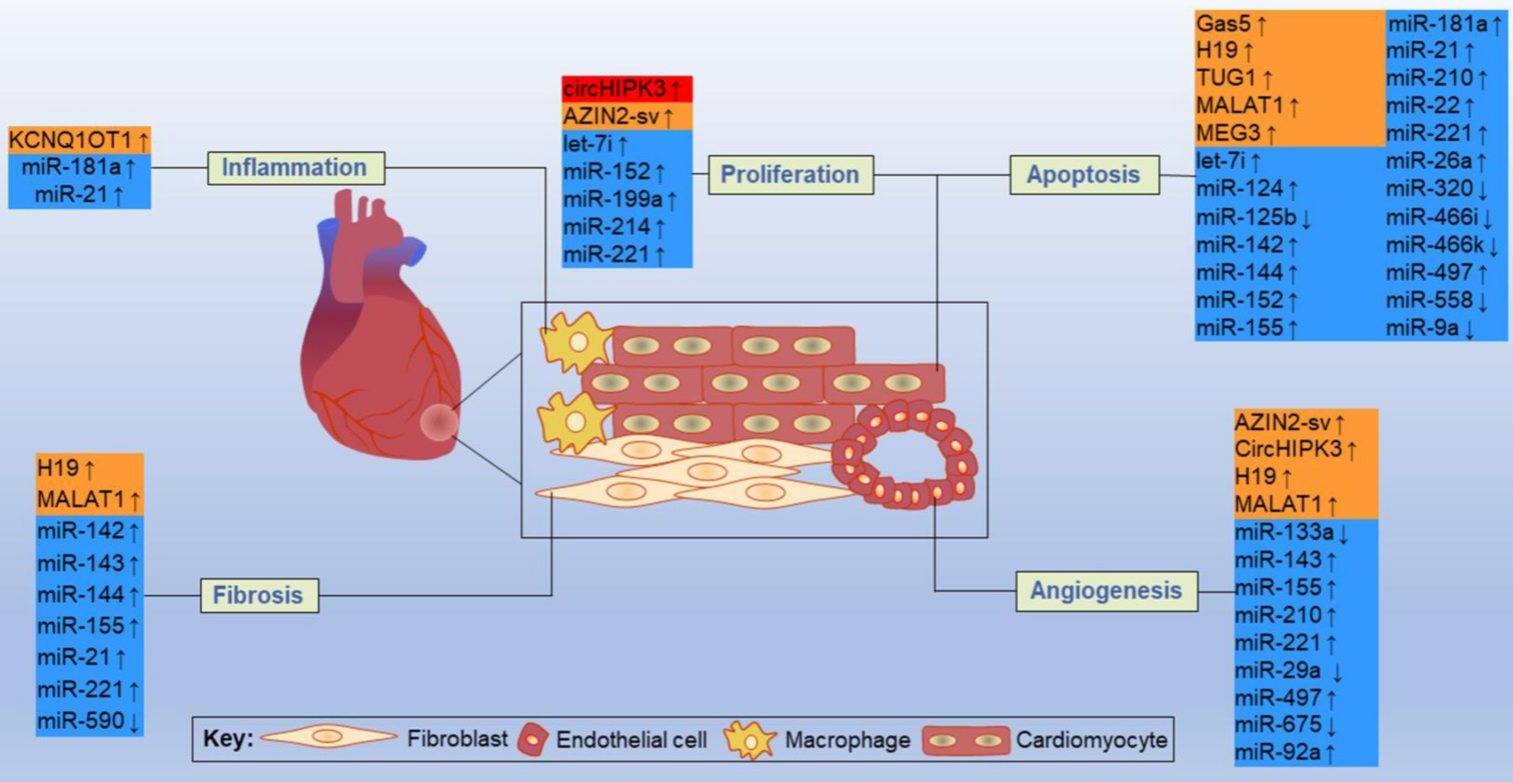
Some representative ncRNAs in the network involved in various functions of the heart after MI are reviewed. The up and down arrows indicate changes in ncRNAs after MI. The red box represents circRNA, the orange boxes represent lncRNAs, and the blue boxes represent miRNAs.

By acting on direct targets, ncRNAs in these networks play various functions in major resident cell types of the heart, such as cardiomyocytes, ECs, fibroblasts and macrophages, and play crucial roles in cell proliferation, apoptosis, fibrosis, inflammation and other processes (**Figure 11**). Inhibition or overexpression of certain ncRNAs or their targets may help alleviate cardiac tissue injury, promote angiogenesis, and prevent cardiac remodeling after MI. However, some ncRNAs may have opposite effects on different targets; therefore, how to rationally utilize these ncRNAs and their targets has become an urgent problem to be solved. Key information, such as carrier selection, treatment time window, treatment dose and possible side effects of ncRNA therapy, needs to be further determined. In short, the interactions between coding and noncoding genes play indispensable roles in the occurrence and development of MI and still deserve great attention from researchers in this field. The rational application of ncRNAs is expected to become a target for the treatment of MI.

## Supplementary Material

Supplementary figure and table.

## Figures and Tables

**Table 1:** List of ncRNA-target and the type of interaction present in networks.

ncRNA	Name	Direct target	Direct effect	PMID	Function*
miRNA	let-7i	FASLG	neg	28350318	anti-apoptosis
miRNA	let-7i	CCND2	neg	30679264	Inhibition of cell proliferation
miRNA	let-7i	E2F2	neg	30679264	
miRNA	miR-124	HIC5	neg	29864957	Pro-apoptosis
miRNA	miR-124	DHCR24	neg	31100313	
miRNA	miR-125b	BAK1	neg	29122578	Anti-apoptosis
miRNA	miR-125b	KLF13	neg	29122578	
miRNA	miR-125b	p53	neg	29921652	
miRNA	miR-125b	p53	neg	30613290	
miRNA	miR-125b	BAK1	neg	30613290	
miRNA	miR-142	APC	neg	32327611	Anti-apoptosis Activation of cardiac fibroblasts
miRNA	miR-142	TP53INP1	neg	31085811	
miRNA	miR-143	IGF-IR	neg	32368046	Pro-angiogenesis
miRNA	miR-143	ERG1	neg	31900593	Inhibition ventricular arrhythmias
miRNA	miR-143	SPRY3	neg	30878395	Pro-fibrosis
miRNA	miR-144	mTOR	neg	30084039	Pro-fibrosis
miRNA	miR-144	PTEN	neg	31737623	Pro-autophagy
miRNA	miR-152	DNMT1	neg	29358670	Promotion of cell proliferation
miRNA	miR-152	p27	neg	29358670	
miRNA	miR-152	ATG12	neg	28350318	
miRNA	miR-155	SOS1	neg	28129114	Anti-fibrosis
miRNA	miR-155	SOCS1	neg	28129114	
miRNA	miR-155	QKI	neg	31191799	Pro-apoptosis
miRNA	miR-155	RAC1	neg	32112145	Anti-angiogenesis
miRNA	miR-155	PAK2	neg	32112145	
miRNA	miR-155	SIRT1	neg	32112145	
miRNA	miR-155	AMPKα2	neg	32112145	
miRNA	miR-181a	STAT1	neg	28597963	Anti-inflammation, Anti-apoptosis
miRNA	miR-181a	c-Fos	neg	28597963	
miRNA	miR-181a	ADAMTS1	neg	32304626	Inhibition of cardiomyocytes hypertrophy
miRNA	miR-199a	HOMER1	neg	28077443	Promotion of cell proliferation
miRNA	miR-199a	HOPX	neg	30125571	
miRNA	miR-21	PTEN	neg	26978580	Anti-apoptosis
miRNA	miR-21	KBTBD7	neg	29991775	Anti-inflammation
miRNA	miR-21	PDCD4	neg	31149048	Anti-apoptosis
miRNA	miR-21	PDCD4	neg	30684569	
miRNA	miR-21	PTEN	neg	30684569	
miRNA	miR-21	STRN	neg	32297634	Disorder of nitrogen metabolism
miRNA	miR-21	SPRY1	neg	33273841	Pro-fibrosis
miRNA	miR-210	CXCR4	neg	29710553	Reduction of hypoxia-induced damage
miRNA	miR-210	AIFM3	neg	32513270	Anti-apoptosis
miRNA	miR-210	EFNA3	neg	28249798	Pro-angiogenesis
miRNA	miR-214	DIO3	neg	27014189	Protection of cardiac systolic function
miRNA	miR-214	PTEN	neg	29584819	Promotion of cell proliferation
miRNA	miR-214	PTEN	neg	30545799	
miRNA	miR-214	GSK3β	neg	30947518	Promotion of cardiac regeneration
miRNA	miR-221	PTEN	neg	32586406	Pro-angiogenesis Anti-apoptosis
miRNA	miR-221	PTEN	neg	32432109	
miRNA	miR-221	FOXO3	neg	32629000	Anti-fibrosis
miRNA	miR-26a	ATM	neg	31990056	Anti-apoptosis, Anti-fibrosis
miRNA	miR-26a	USP15	neg	31512556	Pro-autophagy
miRNA	miR-26a	BAK1	neg	32464547	Anti-apoptosis
miRNA	miR-590	CLIC5	neg	28077443	Promotion of cell proliferation
miRNA	miR-590	HOMER1	neg	28077443	
miRNA	miR-590	ZEB1	neg	31675172	Anti-fibrosis
miRNA	miR-92a	KLF2	neg	32939962	Anti-angiogenesis
miRNA	miR-92a	ATG4A	neg	30571345	Anti-autophagy
miRNA	miR-92a	ABCA8B	neg	30571345	Lipometabolic disturbance
miRNA	miR-92a	CD36	neg	30571345	
lncRNA	AZIN2-sv	miR-214	neg	29584819	Inhibition of cell proliferation
lncRNA	AZIN2-sv	PTEN	pos	29584819	
lncRNA	AZIN2-sv	miR-214	neg	30545799	
lncRNA	AZIN2-sv	TLN1	neg	30545799	Anti-angiogenesis
lncRNA	GAS5	SEMA3A	neg	30099044	Anti-apoptosis
lncRNA	GAS5	miR-142	neg	31085811	Pro-apoptosis
lncRNA	H19	YB-1	neg	31588235	Pro-fibrosis
lncRNA	H19	miR-22	neg	31755219	Reduction of cardiomyocytes damage
lncRNA	H19	miR-675	pos	31119268	Anti-apoptosis, Pro-angiogenesis
lncRNA	KCNQ1OT1	RUNX3	neg	31625414	Pro-inflammation
lncRNA	KCNQ1OT1	DNMT1	pos	31625414	Pro-apoptosis
lncRNA	KCNQ1OT1	miR-466k	neg	32422306	Cardiomyocytes damage
lncRNA	KCNQ1OT1	miR-466i	neg	32422306	
lncRNA	MALAT1	miR-320	neg	29990866	Pro-apoptosis
lncRNA	MALAT1	miR-558	neg	30536615	Anti-apoptosis
lncRNA	MALAT1	miR-497	neg	32393784	
lncRNA	MALAT1	miR-145	neg	30146700	Pro-fibrosis
lncRNA	MALAT1	miR-92a	neg	32939962	Pro-angiogenesis
lncRNA	MEG3	FUS	pos	30287867	Pro-apoptosis
lncRNA	MEG3	p53	pos	31631486	
lncRNA	TUG1	miR-124	neg	29864957	Anti-apoptosis
lncRNA	TUG1	miR-9a	neg	31787746	Pro-apoptosis
circRNA	circHIPK3	miR-17	neg	31595165	Increase of Ca^2+^ in cardiomyocytes
circRNA	circHIPK3	N1ICD	pos	32736292	Promotion of cell proliferation
circRNA	circHIPK3	miR-133a	neg	32736292	Pro-angiogenesis
circRNA	circHIPK3	miR-29a	neg	32733638	

* Function refers to the pathophysiological changes after MI caused by upregulation of the ncRNA expression.

## Abbreviations

ceRNA: competing endogenous RNA
circRNAs: circular RNAs
ECs: endothelial cells
lncRNAs: long noncoding RNAs
MI: myocardial infarction
miRNAs: microRNAs
ncRNAs: noncoding RNAs
