# Supplementary material for: The Networks of Noncoding RNAs and Their Direct Molecular Targets in Myocardial Infarction
Source: Int J Biol Sci. 2022 May 1;18(8):3194–208. doi: 10.7150/ijbs.69671 (PMC9134914; doi:10.7150/ijbs.69671)
Supplement: Supplementary file 1 — Supplementary figure and table. [file ijbsv18p3194s1.pdf]

Table S1. The Retrieval Strategy of the literatures related to non-coding RNAs and myocardial infarction in the past five years.

**Retrieval strategy**	**Items found**
(("Myocardial Infarction"[Mesh]) OR ("Myocardial Infarction")) AND (((((microRNA) OR (miRNA)) OR (ncRNA)) OR ("non coding RNA")) OR (lncRNA))	1526
**Time Span:** 2016-2020	
**The Last Retrieval Time:** August 27, 2021	

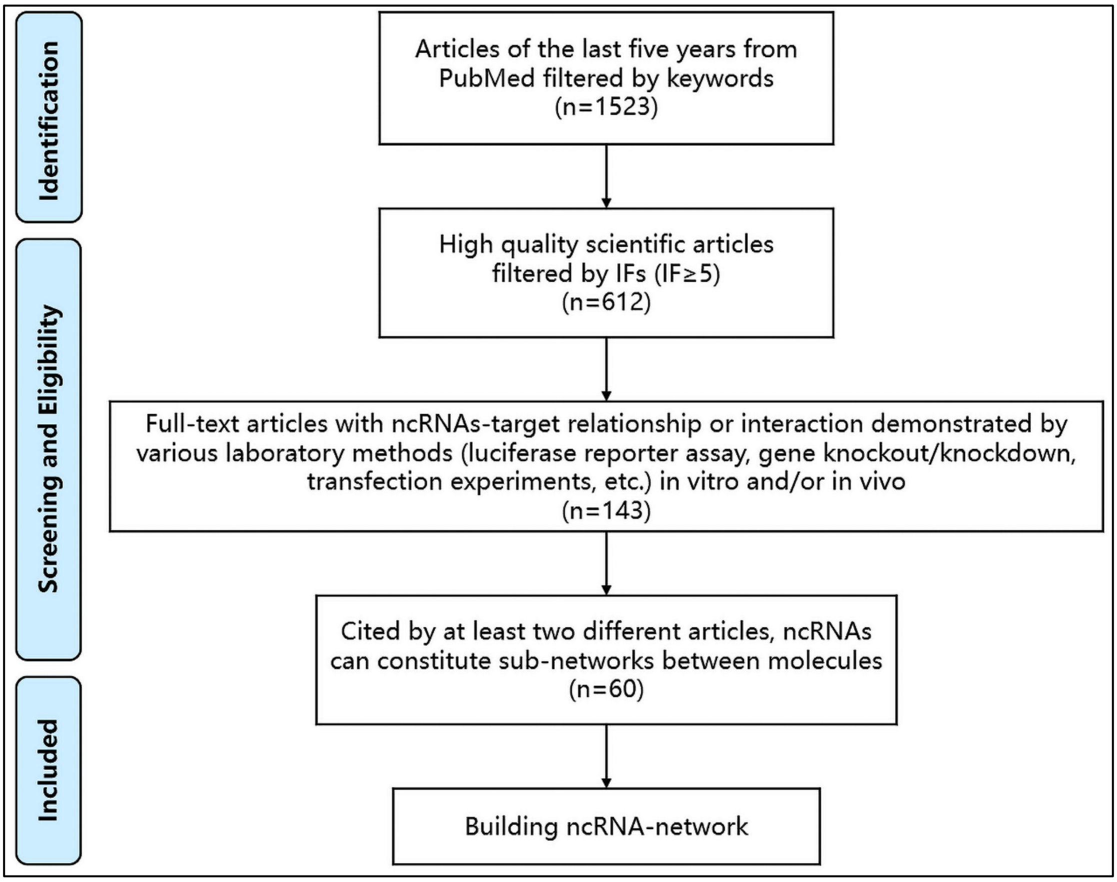

Figure S1. The flowchart outlining the results of the screening and selection process.
